# Opto-mechanically generated resonant field enhancement

**DOI:** 10.1038/s41598-022-22987-3

**Published:** 2022-10-31

**Authors:** Alicia Fresno-Hernández, Manuel I. Marqués

**Affiliations:** 1 Grupo de Displays y Aplicaciones Fotónicas (GDAF), Universidad Carlos III de Madrid (UC3M), 28911 Leganés, Madrid Spain; 2 Departamento Física de Materiales, IFIMAC and Instituto Nicolás Cabrera, Universidad Autónoma de Madrid (UAM), Ciudad Universitaria de Cantoblanco, C. Francisco Tomás y Valiente, 7, 28049 Madrid, Spain

**Keywords:** Nanoparticles, Nanophotonics and plasmonics, Optical manipulation and tweezers

## Abstract

A link between the resonant cumulative field enhancement experienced by a chain of plasmonic nanoparticles in a light field and the orientation of the chain with respect to the field is obtained. We calculate analytically the optical torque and the equilibrium configuration and we show how stable orientations are triggered by the geometric resonance conditions. Analytical predictions are checked using numerical calculations based on the coupled dipoles method (CDA) for the particular case of a chain of silver nanoparticles. The reported resonance driven optical torque allows for a tuning of the orientation of the chain depending on radiation’s wavelength.

## Introduction

Techniques like surface enhanced Raman scattering (SERS), fluorescence or non-linear optics take advantage of the enhanced local fields that arise in the vicinity of plasmonic nanoparticles. This field enhancement is further increased when considering arrays of several particles or dimers^1–12^. One example is the so called cumulative field enhancement effect^13^ taking place in one dimensional arrays of nanoparticles illuminated in end-fire configuration (array parallel to the radiated energy). This phenomenon is based on the constructive interference from all fields coming from the nanoparticles and producing a large field enhancement at the extremity of the chain.

For field enhancements based on multiple interactions among several nanoparticles, the orientation of the array with respect to the propagation of the excitation wave is crucial. One may wonder if resonant configurations inducing field enhancement may be auto-stabilised by the opto-mechanical interactions taking place between the array of nanoparticles and the electromagnetic field.

The possibility to manipulate the position and orientation of small particles by using optical forces and torques is nowadays a reality which has experienced a great development during last decades. Optical forces are used to cool^14,15^ , trap^16^ neutral atoms and biological systems, to rotate small objects^17^ and even to translate or/and rotate metallic nanowires^18–20^. A lot of powerfull manipulation techniques are based in optical tweezers^16^, which are single-beam optical traps that use the forces exerted by a strongly-focused beam of light to trap small particles.

The arrays of nanoparticles may be created also by optical forces using different techniques like, for example, shaped light fields^21^, optical printing^22^, using the forces generated by the phase gradients of light^23^, creating optofluidic potential wells^24^, with single rapidly scanned optical tweezers^25–27^, or by pointing a laser at a diffraction grating (which could be created, for example, with a computer-generated hologram^28–30^) generating multiple optical traps^28,31^. Once in place, tweezers-organized structures can be fixed by gelling in order to create permanent structures with functionality. When created, the arrays may be further manipulated promoting propulsion of the array by torque-less radiation pressure^32^ or increasing/decreasing its diffusion in a optical lattice structure^33^.

The aim of this paper is to analyze the use optical forces and torques to rotate an already existing array of plasmonic nanoparticles in order to induce a specific configuration promoting field enhancement. Or, equivalently, to analyze if different wavelengths can be used to rotate the array of nanoparticles into a specific resonant angle with respect to the incoming radiation.

## Geometric resonances

**Figure 1:**
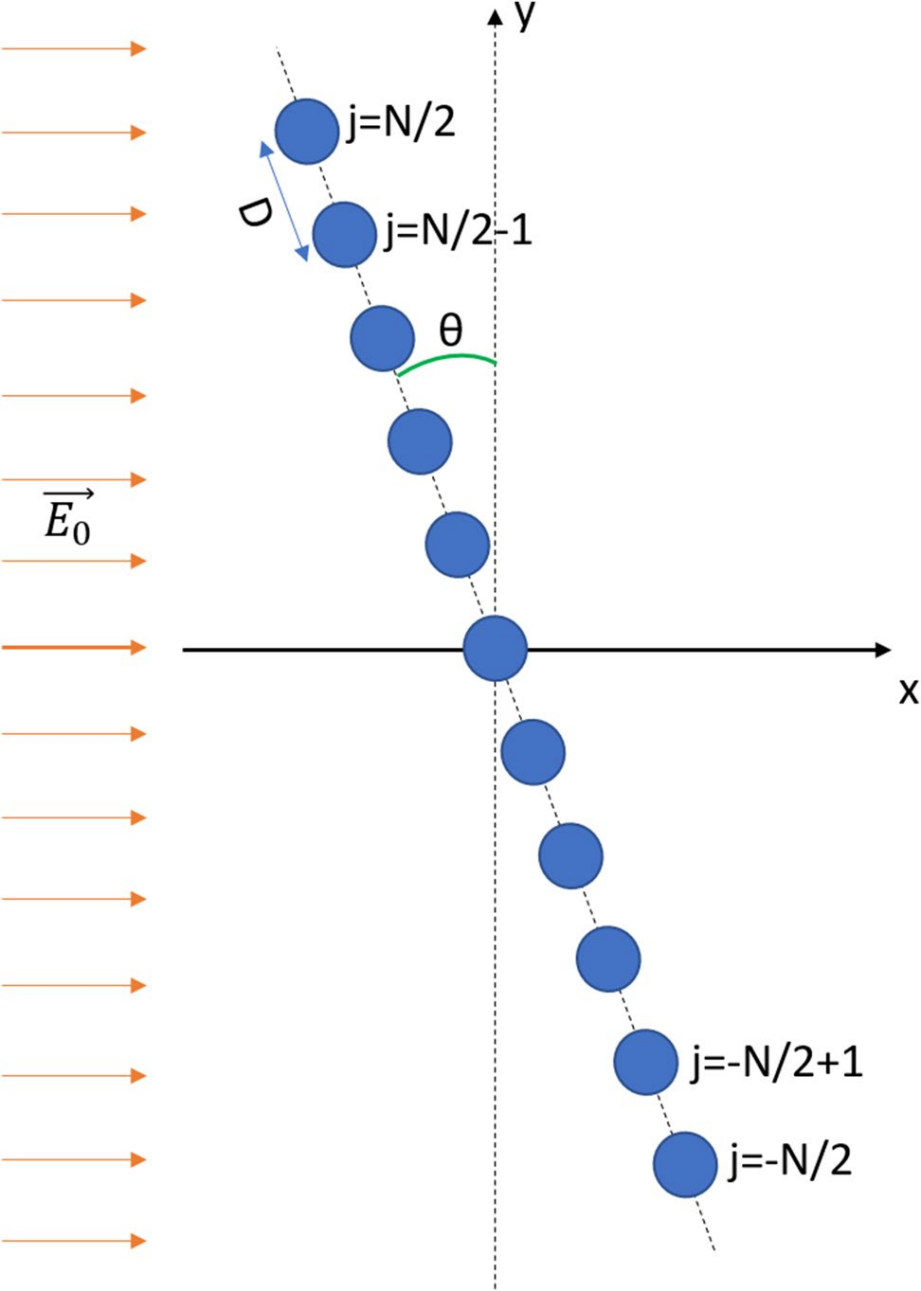
The system analyzed consists on a chain of N + 1 nanoparticles free to rotate an angle $\theta$ around the Z axis. The array is illuminated with a plane wave, Z-polarized, and propagating in the X direction. By considering each nanoparticle as a single electric dipole, the torque on the system and the equilibrium configurations are calculated using multiple scattering calculations.

First, we analyze in detail the origin of the geometric resonances and the cumulative field enhancement reported for arrays of plasmonic nanoparticles^13^. In particular, we analyze a system made up of a chain of $N+1$ electric dipoles with polarizability $\alpha$, separated by a distance *D* an located in the $X-Y$ plane (see Fig. 1). The position of each dipole is given by $x_j=-jDsin(\theta )$ and $y_j=jDcos(\theta )$ being $\theta$ the angle form by the chain an the *Y* axis, and *j* ranging from $-N/2$ to *N*/2. The system is illuminated with an electromagnetic wave traveling in the *X* direction. For the sake of simplicity we consider a plane wave polarized in the *Z* direction and propagating on air with a refractive index equal to one.

The total field on each dipole $p_j$ is given by

1 $$\begin{aligned} E_j=E_0e^{-ikjDsin(\theta )}+k^2\alpha \Sigma _{m \ne j} G_{mj}E_m \end{aligned}$$

Being $k=2\pi /\lambda$ the wave vector, $E_0$ the field’s amplitude and $G_{mj}$ the Green tensor for particles *m* and *j*. We now consider two simplifications: First a single scattering approach

2 $$\begin{aligned} E_m=E_0e^{-ikmDsin(\theta )} \end{aligned}$$

and, secondly, a far field approximation

3 $$\begin{aligned} G_{mj}=\frac{e^{ik|m-j|D}}{4\pi |m-j|D}. \end{aligned}$$

Within these approaches the field on particle *j* is given by

4 $$\begin{aligned} E_j=E_0e^{-ikjDsin(\theta )} \left(1+\frac{k^2}{4\pi D}[S_1+S_2] \right) \end{aligned}$$

being $S_1$ the contribution from all dipoles $p_m$ with $m<j$ and $S_2$ the contribution from all dipoles $p_m$ with $m>j$. These sums are given by

5 $$\begin{aligned} S_1&= \sum \limits_{p=1}^{j+N/2} \alpha \frac{e^{ikDp(1+sin(\theta ))}}{p}(1-\delta _{j,-N/2}) \end{aligned}$$

6 $$\begin{aligned} S_2&=\sum \limits_{p=1}^{N/2-j} \alpha \frac{e^{ikDp(1-sin(\theta ))}}{p}(1-\delta _{j,N/2}) \end{aligned}$$

with $p=|m-j|$. In general, due to the null averaged value of sines and cosines, these sums have a small contribution compared to the external field ($S_1 \sim 0, S_2 \sim 0)$. However for a chain parallel to the *k* vector ($\theta =\pi /2$, end-fire configuration) the contribution of the dipoles with $m>j$ is given by

7 $$\begin{aligned} S_2(\theta =\pi /2)=\alpha \sum \limits_{p=1}^{N/2-j} \frac{1}{p}=\alpha H(N/2-j) \end{aligned}$$

being *H* the harmonic number. Note, how all dipoles with $m>j$ contribute constructively to the field. This is the so called cumulative field enhancement effect^13^. This geometric resonance does not depend on $\lambda$ and is due to the path compensation taking place for all dipoles with $m>j$ and not occurring for the dipoles with $m<j$. If $\theta =-\pi /2$ we have the same effect, but the contribution is now from all dipoles with $m<j$

8 $$\begin{aligned} S_1(\theta =-\pi /2)=\alpha \sum \limits_{p=1}^{j+N/2} \frac{1}{p}=\alpha H(j+N/2) \end{aligned}$$

It is also possible to find geometric resonance angles depending on the radiation wavelength. They are given by $kD(1+sin(\theta ))=2\pi$ and $kD(1-sin(\theta ))=2\pi$ (other resonances for integer values of $2\pi$ are also possible). For the first angle, only the dipoles with $m<j$ contribute

9 $$\begin{aligned} S_1(\theta =asin(\lambda /D-1))=S_1(\theta =-\pi /2) \end{aligned}$$

and for the second angle, only the dipoles with $m>j$ contribute

10 $$\begin{aligned} S_2(\theta =asin(1-\lambda /D))=S_2(\theta =\pi /2) \end{aligned}$$

These geometric resonances may be understood also from the point of view of optical path compensation. For example, for an angle $\theta >0$, the total phase of the scattered wave at a dipole *j* coming from a dipole located at $m=j-p$ (with $p>0$) is given by the phase of the plane wave at the scatter, ($-kDsin(\theta )(j-p)$) plus the phase acquired by the wave when traveling from *m* to *j*, (*pkD*). Then, the total phase is given by $-jkDsin(\theta )+pkD(1+sin(\theta ))$, which does not depend on *p* if $kD(1+sin(\theta ))=2\pi$. For this value, the extra phase achieved by the wave at the scattered *m* is compensated by the phase accumulated along the path joining dipole *m* with dipole *j*. When $\theta =0$, the incoming field has the same phase on all dipoles and, for that reason, the requirement for optical path compensation is $D=\lambda$. Note how, for wavelengths $\lambda > 2D$ the only possible solution for the equation $kD(1\pm sin(\theta ))=2\pi$ is $\theta =\mp \pi /2$, implying that it is impossible to achieve path compensation for orientations different from the end-fire configuration. On the other hand, as already mentioned, for $\lambda <D$ there are multiple resonant solutions given by the equation $kD(1\pm sin(\theta ))=n2\pi$ with $n=1,2,3,...$. Therefore, for a value of *kD*, optical path compensation may be obtained for different orientation angles given by $sin(\theta )=\pm (n\lambda /D-1)$.

In short, we may find the cumulative field enhancement effect for angles not corresponding with the end-fire configuration and depending on the relation between wavelength and separation distance of the dipoles in the chain.

## Optical torque and resonant equilibrium configurations

We now consider that the array of dipoles is free to rotate in the $X{-}Y$ plane around a Z-axis located at the middle of the chain. The system has a single degree of freedom given by $\theta$. In order to obtain the equilibrium configurations we must calculate the optical torque and find the angles with zero value of the torque. Next, we will show how the equilibrium arrangements are promoted by resonant configurations.

The torque is given by

11 $$\begin{aligned} T=\sum \limits_{j=-N/2}^{j=N/2} |j|DF_\theta (j) \end{aligned}$$

being $F_\theta (j)$ the polar component of the optical force in the *j* particle. Since we are dealing with electric dipoles, the polar component of the force is given by^34^

12 $$\begin{aligned} F_\theta (j)=\frac{\varepsilon _0}{2}\Re \left(\alpha E_j \frac{\partial E_j^*}{r\partial \theta } \right) \end{aligned}$$

being $r=|j|D$. Taking into account Eq. (1) and the radial character of the Green tensor in the far field, we obtain:

13 $$\begin{aligned} F_\theta (j)=\frac{\varepsilon _0}{2|j|D}\Re \left[\alpha E_{j}\frac{\partial E_j^*}{\partial \theta } \right] \end{aligned}$$

with

14 $$\begin{aligned} \frac{\partial E_j^*}{\partial \theta }=ikjDcos(\theta )E_0 e^{ikjDsin(\theta )} \end{aligned}$$

With the force, we can calculate the optical torque in the system

15 $$\begin{aligned} T=\sum \limits_{j=-N/2}^{j=N/2}\frac{\varepsilon _0E_0^2cos(\theta )k^3j}{8 \pi }\Re [i \alpha (S_1+S_2)] \end{aligned}$$

At the cumulative enhancement field resonant conditions given by $\theta =\pm \pi /2$ the polar components of the force are equal to zero and, then, the torque is also equal to zero. So, the chain has an equilibrium configuration at the end-fire configuration, independently of radiation’s wavelength.

Now, we analyze if the wavelength dependent resonant configurations previously reported also correspond to an equilibrium configuration with zero value of the torque. For configurations close to the resonant condition ($sin(\theta ) \sim \lambda /D -1$), we have $S_2 \sim 0$ but $S_1 \ne 0$ and then, the torque near the resonant condition ($T_{RC}$) is given by

16 $$\begin{aligned} T_{RC}=-\kappa \sum \limits_{j=-N/2}^{j=N/2} \sum \limits_{p=1}^{j+N/2}\frac{j}{p}[\Re (\alpha ^2)sin(kDp(1+sin(\theta )))+\Im (\alpha ^2)cos(kDp(1+sin(\theta )))] \end{aligned}$$

with

17 $$\begin{aligned} \kappa =\frac{\varepsilon _0 E_0^2 cos(\theta ) k^3}{8 \pi } \end{aligned}$$

The torque may be rewritten in a more convenient way as (see S.I.):

18 $$\begin{aligned} T_{RC}=-\kappa \sum \limits_{p=1}^{N}\frac{N-P+1}{2}[\Re (\alpha ^2)sin(kDp(1+sin(\theta )))+\Im (\alpha ^2)cos(kDp(1+sin(\theta )))] \end{aligned}$$

The values of summations above are all known (see S.I.) and then, taking into account that near resonance $cos(kD(1+sin(\theta ))\sim 1$, we obtain the following value for the torque near the resonant condition

19 $$\begin{aligned} T_{RC}=-\frac{\kappa }{8}csc^{2} \left(\frac{kD(1+sin(\theta ))}{2}\right)(-\Im (\alpha ^2)T_1+\Re (\alpha ^2)T_2) \end{aligned}$$

being

20 $$\begin{aligned}&T_1=cos(kD(1+sin(\theta ))(N+1))-1 \end{aligned}$$

21 $$\begin{aligned}&T_2=-sin(kD(1+sin(\theta ))(N+1))+(N+1)sin(kD(1+sin(\theta ))) \end{aligned}$$

Once the torque in known we calculate the equilibrium configuration near the geometric resonance by establishing $T_{RC}=0$ at $kD(1+sin(\theta ))=2\pi +\Delta$, which implies the following equation for $\Delta$

22 $$\begin{aligned} \frac{\Im (\alpha ^2)}{\Re (\alpha ^2)}[cos(\Delta (N+1))-1]+sin(\Delta (N+1))=(N+1)\Delta \end{aligned}$$

This equation is solved numerically and, for $-10<\Im (\alpha ^2)/\Re (\alpha ^2)<10$ we obtain:

23 $$\begin{aligned} \Delta \sim -\frac{3.55}{(N+1)}atan \left(\frac{3\Im (\alpha ^2)}{4\Re (\alpha ^2)}\right) \end{aligned}$$

The final value of the equilibrium angle with null torque is then given by

24 $$\begin{aligned} \theta =asin \left(\frac{\lambda }{D}-1+\frac{\lambda }{2\pi D}\Delta \right) \end{aligned}$$

Note how, as the system gets larger ($N \rightarrow \infty )$ the parameter $\Delta \rightarrow 0$ and then, the equilibrium configuration tends to the wavelength dependent geometric resonance promoting cumulative field enhancement. The same conclusion is reached if the analysis is repeated for the other geometric resonance given by $\sin (\theta )=1-\lambda /D$.

To summarize, we have proved that the resonant orientations, leading to enhancement of the fields, are auto-established by the optical torque generated on the array of nanoparticles. Next, we will use discrete dipole numerical simulations in order to check if these analytical predictions hold on specific electric dipoles arrays with no approximations assumed (i.e. multiple scattering effects and interactions in the short, middle and long ranges will be taken into account).

## Numerical analysis of the torque for an array of silver nanoparticles

The system proposed is shown in Fig. 1. It consists on a chain made up of N+1=51 silver nanoparticles that is free to rotate an angle $\theta$ around the Z-axis. All nanoparticles are located in the $X{-}Y$ plane, and are illuminated with a plane wave polarized in the Z-axis and whose direction of propagation is the X-axis. Therefore, the expression for the electric field is given in Eq. (25), where $\overrightarrow{k}=(k_x,0,0)$ is the wave vector and $E_0$ is the amplitude.

25 $$\begin{aligned} \overrightarrow{E_0}=E_0\cdot e^{i(\overrightarrow{k}\overrightarrow{r}-wt)} \end{aligned}$$

Silver nanoparticles are considered as electric dipoles with polarizability given by Eq. (26), being $\alpha _0$ the static polarizability, which in our case will be obtained from the permittivity, by making an interpolation of the Johnson & Christy database.

26 $$\begin{aligned} \alpha =\frac{\alpha _0}{1-i\alpha _0\frac{k^3}{6\pi }} \end{aligned}$$

In particular, we analyze silver particles of 50nm radius in vacuum, separated by a distance $D=498\;\text{nm}$. Therefore, our particles are placed equidistant and with a fixed distance, which implies that, at the time of a real experiment, they may be fixed on a solid matrix or substrate, which for simplicity of the simulation we are assuming to have the same refractive index as the background. A detailed description of the coupled dipole method (CDA) used to calculate the torque as a function of the angle $\theta$ is shown in the SI.

**Figure 2:**
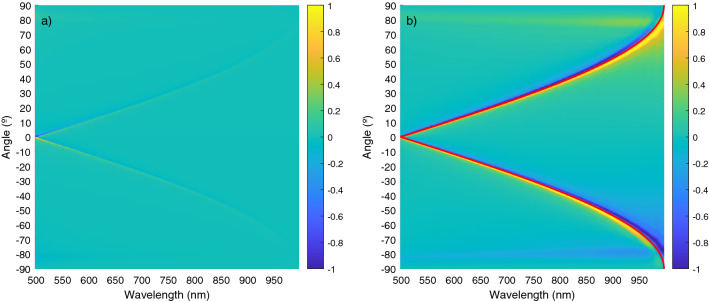
Color map of the torque versus wavelength and orientation angle. Yellow regions corresponds to positive torques (anti-clock wise oriented), blue regions to negative torques (clock wise oriented). In (**a**) The torque is normalized to the maximum absolute value. In (**b**) the torque is normalized to the maximum value for each wavelength. The resonant angle promoting cumulative field enhancement is marked with a red line.

We consider an angle $\theta$, varying from − 90$^\circ$ to 90$^\circ$ in 10,000 steps. In Fig. 2 the plots of the torque as a function of angle and wavelength (which, in this case, will vary from 498 to 996 nm in steps of 1nm) are shown in a color map. We also plot the value of the torque normalized to the maximum value for each wavelength.

**Figure 3:**
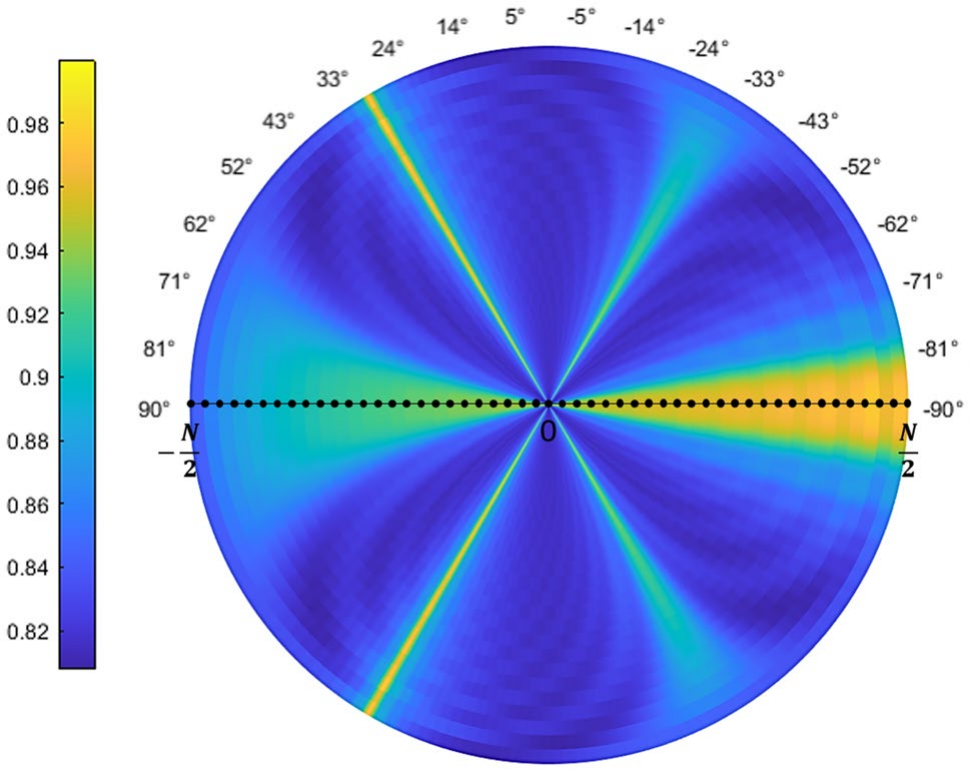
Polar color map of the normalized value for the intensity on each particle as the array rotates from $-90$ to 90 for a wavelength of 750 nm. Note how the field is increased at the end fire configuration ($\theta =\pm \pi /2$) and at the resonant angle given approximately by $\pm 30$°.

The equilibrium orientations, with zero torque ($T=0$) and negative slope ($\partial T/ \partial \theta < 0$) are given by the green colored values located between yellow (positive torque for angles smaller than the equilibrium angle) and blue (negative torque for angles larger than the equilibrium angle) regions. In Fig. 2 we have plotted, together with the color map, a red line corresponding to the resonant orientations given by $sen\theta =\pm \left( \frac{\lambda }{D}-1\right)$. Note how the resonant orientations are very close to the equilibrium configurations of the system. The other, wavelength independent, resonant values with $\theta = \pm \pi /2$ also correspond to equilibrium configurations although, in this case, the stiffness of the orientation trapping is much smaller. Note how these figures predict a wavelength dependent stability angle so, by changing the wavelength with respect to the nanoparticles distance, we can tune the orientation of the one dimensional array of nanoparticles and, more importantly, this angle corresponds to the resonant orientation where cumulative enhancement of the field at the final particle takes place. This enhancement of the field is explicitly shown in Fig. 3 where we present a polar plot of the intensity of the field on each particle, as the system rotates, for the particular case of $\lambda =750\;\text{nm}$. Field enhancement takes place close to 30°.

**Figure 4:**
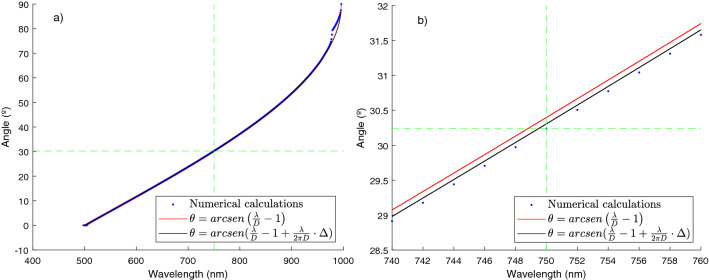
(**a**) Analytical prediction of the equilibrium angle versus wavelength (black line) and value obtained numerically (blue points). The resonant angle is plotted with a red line. In (**b**) a zoom for wavelengths ranging from 740 to 760 nm is shown. Green line marks the expected equilibrium value for a wavelength of 750 nm, close to 30°.

To be more precise, we have numerically calculated the equilibrium points where the torque is zero and, in addition, has the largest negative slope. The results (blue points) can be seen in Fig. 4 compared to the resonant angle (red line) and the equilibrium angle expected from our analytic calculation (black line). If we zoom in the graph between 740 and 760 nm, we can appreciate how our prediction is very close to the numerical value (differences come mainly from the single scattering approximation) and slightly differs from the resonant value. Note how, for $\lambda =750\;\text{nm}$, equilibrium is obtained at $\sim 30^{\circ }$, (marked with a green dotted line) corresponding closely with the value of the angle promoting field enhancement (see Fig. 3).

**Figure 5:**
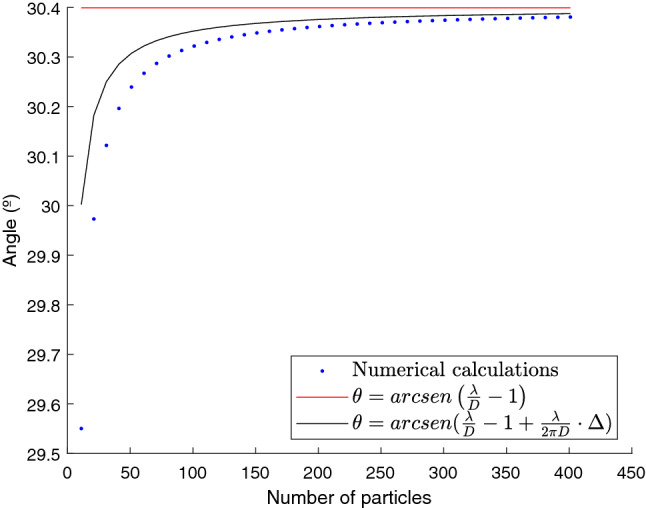
Equilibrium angle versus number of particles in the array for a wavelength of 750 nm. Values obtained with the numerical calculations are plotted in blue. Black line is the value obtained from the analytical prediction (Eq. 24) and the red line marks the resonant value. Note how all three values converge for a large number of particles.

**Figure 6:**
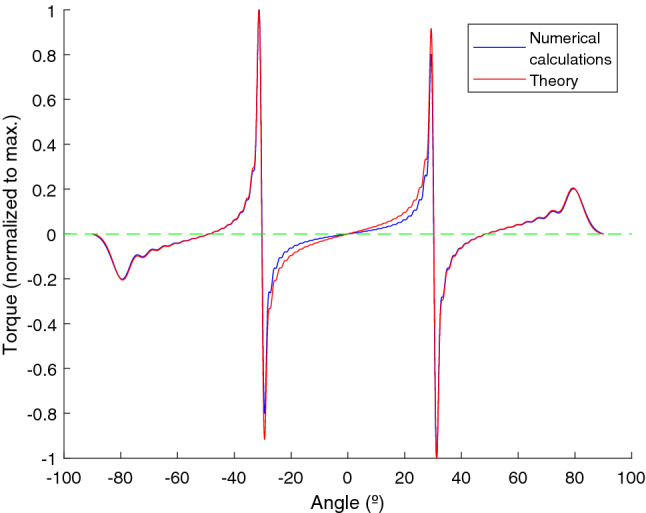
Normalized optical torque versus angle for a wavelength of 750 nm and $N=51$ particles. The torque is obtained analytically (red line) using Eq. (15) and numerically (blue line) using CDA calculations.

**Figure 7:**
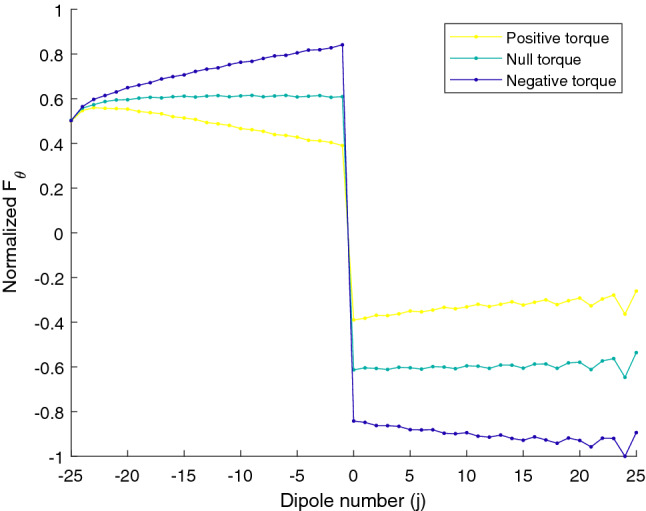
Normalized polar component of the force on each particle for the maximum positive torque, maximum negative torque and zero torque in the resonance region corresponding to $sin(\theta )=\lambda /D-1$ with $\lambda =750 \; \text{nm}$.

Our theoretical analysis predicts an asymptotic approach of the equilibrium angle to the resonant value as the size of the chain increases. This behavior is tested in Fig. 5, where we plot the equilibrium angle versus number of particles in the chain. Note how, as the number of particles increases, the asymptotic approach to the resonant value is obtained. For completeness, in Fig. 6, we have compared the expression for the torque (Eq. 15) with the numerical value. Excluding small deviations, coming mainly from the single scattering approach, the behavior of the optical torque is correctly predicted and, more importantly, the equilibrium angles, with zero value of the torque, are reproduced. Finally, to get a more complete picture of the behavior of the torque, we have calculated, for $\lambda =750\;\text{nm}$ and in the region close to 30°, the polar components of the force in the dipoles for the orientation with maximum positive torque, maximum negative torque and for zero torque. Results are shown in Fig. 7 Note how, for zero torque, the forces on both sides of the chain tend to be compensated while, for the positive torque, forces at $j<0$ are larger (in absolute value) than the forces at $j>0$ and, for negative torque, absolute value of the forces at $j>0$ are larger than the absolute value of the forces at $j<0$.

## Numerical analysis for wavelengths below inter-particle distance

**Figure 8:**
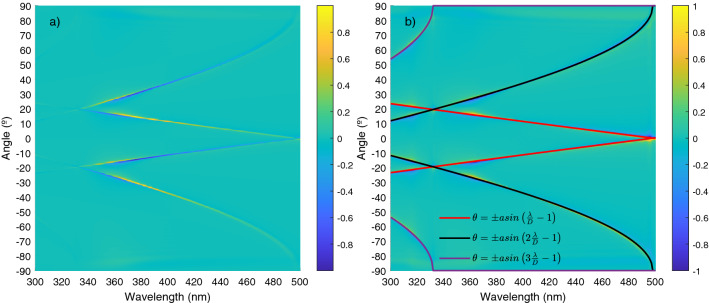
Color map of the torque versus wavelength and orientation angle, for a range of wavelengths between 300 and 500 nm. Yellow regions corresponds to positive torques (anti-clock wise oriented), blue regions to negative torques (clock wise oriented). In (**a**) the torque is normalized to the maximum absolute value. In (**b**) the torque is normalized to the maximum value for each wavelength. The resonant angles for different diffraction orders promoting cumulative field enhancement are marked with coloured lines (in red the first diffraction order, in black the second order and in purple the third order).

All previous analysis has been made in a range of wavelengths ranging from D to 2D. However, it is also interesting to analyze wavelengths between 300 and 500 nm because, in this range, the plasmonic resonance of the silver nanoparticles takes place and the real part of the polarizability turns negative between 316 and 354 nm approximately. We plot in Fig. 8a,b the color map of the torque versus wavelength and orientation angle for this range of wavelengths. The first noticeable feature is the appearance of several equilibrium branches or equilibrium angles per wavelength. As explained before, these branches are due to the new diffraction orders appearing at lower wavelengths for $n=2$ and 3. Our calculations show how equilibrium configurations with zero torque also emerge at these higher orders, allowing for the existence of multiple equilibrium angles at a given wavelength.

**Figure 9:**
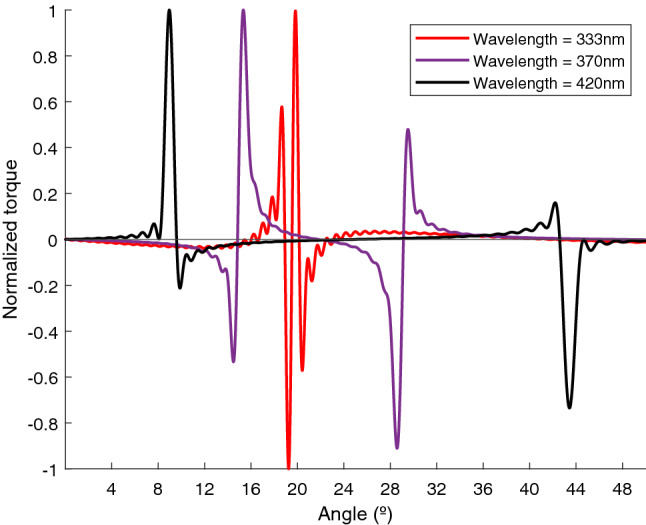
Normalized torque versus angle for $\lambda$ equal to 333 nm (red line), 370 nm (purple line) and 420 nm (black line).

However, not all zero torques at the geometric resonances correspond with stable configurations. In particular, we have considered three different wavelengths within the resonance region corresponding to three different possibilities: 420 nm ($\Re (\alpha ) > \Im (\alpha )$), 370 nm ($\Im (\alpha ) > \Re (\alpha )$ and $\Re (\alpha ) > 0$) and 333 nm ($\Im (\alpha ) > \Re (\alpha )$ and $\Re (\alpha ) < 0$) and we have plotted the torque versus angle at positive angles for the three cases in Fig. 9. Note how, for the largest wavelengths (420 nm) the torque at equilibrium has a negative slope. This is the same result we have already reported in previous sections corresponding with an stable configuration. Then, we may deduce that if $\Re (\alpha ) > \Im (\alpha )$ (basically out of plasmonic resonance regions) the resonant configurations are stable. The same stable case is found for the lower wavelength (333 nm) within the resonance region. Actually, in this case, the two resonance branches are extremely close but they both generate stable states. So, we may infer that if $\Im (\alpha ) > \Re (\alpha )$ but $Re(\alpha ) <0$ the resonant configurations are also stable. However, for the intermediate wavelength with $\Im (\alpha ) > \Re (\alpha )$ and $Re(\alpha ) >0$ the slopes of the torque are positive in the resonant equilibrium angles and then, the equilibrium states are unstable. To sum up, from these numerical results we may propose that, out from the plasmon resonant region (i.e. in regions with $\Re (\alpha ) > \Im (\alpha )$) all resonant equilibrium states are stable and, within the plasmon resonance region, they are stable as long as the real part of the polarizability is smaller than zero. In order to understand the reason for this behavior and the origin of the zero torque equilibrium states, we must analyze the system within the near resonance region.

## Extensive contribution in the near-geometric resonance

In order to understand the physical mechanism allowing for the existence of this equilibrium configurations it is very helpful to analyze the behavior of the scattered fields near the geometric resonance. Let us consider an orientation angle $\theta$, very close to one of the geometric resonant angles, given by:

27 $$\begin{aligned} kD(1+sin(\theta ))=2\pi +\delta \end{aligned}$$

with $$\delta$$ fulfilling

28 $$\begin{aligned}&cos(kDp(1+sin(\theta ))\sim 1 \end{aligned}$$

29 $$\begin{aligned}&sin(kDp(1+sin(\theta )) \sim p\delta \end{aligned}$$

for any value of p ranging from 1 to *N*.

For a particle *j*, at this angle, $S_2 \sim 0$ and $S_1$ is given by

30 $$\begin{aligned} S_{1}=\alpha \sum \limits_{p=1}^{j+N/2} \left(\frac{1}{p}+i\delta \right)=\alpha \left(H \left (j+\frac{N}{2}\right)+i\delta \left(j+\frac{N}{2}\right)\right) \end{aligned}$$

Note how, apart from the harmonic contribution already reported in Eq. (8), there is also an extensive contribution in the near-geometric resonance region. This an addition in which all particles with $p<j+N/2$ contribute equally, i.e., the 1/*p* distance decay effect is compensated by the behavior of the phase, proportional to *p*. In principle, due to the negligible value of $\delta$, this addition is very small when compared with the harmonic contribution and, in our case, has no influence in the intensity near the resonance angle. However, this term plays a fundamental role in the behavior of the optical torque.

In the particular case we are now considering ($S_2\sim 0$), the torque near resonance has the value (see Eq. 15):

31 $$\begin{aligned} T_{RC}=- \sum \limits_{j=-N/2}^{j=N/2}j\kappa \left[\Im ({\alpha ^2})H \left(j+\frac{N}{2}\right)+\Re (\alpha ^2)\delta \left(j+\frac{N}{2}\right) \right] \end{aligned}$$

For the exact geometric resonant angle ($\delta =0$) the harmonic cumulative effect makes the torque different from zero (negative). However, very near the resonance angle and due to the extensive contribution, it is possible to find a value of $\delta =\Delta$ fulfilling

32 $$\begin{aligned} \Delta =-\frac{\Im (\alpha ^2)\Sigma _{j=-N/2}^{j=N/2}jH(j+N/2)}{\Re (\alpha ^2)(\frac{N^3}{12}+\frac{N^2}{4}+\frac{N}{6})} \end{aligned}$$

and making $T_{RC}=0$ (to obtain the above expression we have used $\Sigma _{j=-N/2}^{j=N/2}j(j+N/2)=N^3/12+N^2/4+N/6$).

The values inferred for the equilibrium angle with this value of $\Delta$, if plotted in Fig. 4, are not distinguishable from the ones already reported. Note how again, as the system gets larger ($N \rightarrow \infty )$, the parameter $\Delta \rightarrow 0$.

To sum up, the existence of a zero torque equilibrium configuration near resonance is due to the extensive contribution to the scattered fields. At $\delta =\Delta$ the harmonic cumulative contribution to the torque is canceled by the extensive term. Since $T_{RC} <0$ at $\delta =0$, an stable equilibrium is attained only if $\Delta <0$. In consequence, we may determine the stable equilibrium condition as $\Im (\alpha ^2)/\Re (\alpha ^2) > 0$ which corresponds to ($\Re (\alpha )>\Im (\alpha )$) or ($\Re (\alpha )<\Im (\alpha )$ and $\Re (\alpha )<0)$. This result explains our numerical findings and, in particular, justifies why stable equilibrium is no longer attained near the plasmon resonance of the silver nanoparticles, in the regions with $\Re (\alpha )>0$.

## Conclusions

We have demonstrated that the equilibrium orientations of a one dimensional large array of plasmonic nanoparticles illuminated with a plane wave and free to rotate around a central axis are given by the resonant configurations promoting cumulative field enhancement. The zero torque configurations found are due to the existence of an extensive contribution to the scattered fields taking place in the near resonance region. We have obtained an analytic expression for the equilibrium angle and we have related it with the resonant values previously described. Our predictions have been checked using discrete dipole calculations with no approximations assumed. The applications of these resonant configurations, auto-stabilised by the opto-mechanical interactions taking place between the array of nanoparticles and the electromagnetic field, are twofold; on the one hand, the enhancement of the field near the particles and, on the other hand, the possibility to rotate the array of nanoparticles into a specific resonant angle given by the frequency of the external field.

## Supplementary Information

Supplementary Information.

## Data Availability

The datasets used and/or analysed during the current study are available from the corresponding author on reasonable request.
