# Supplementary material for: Opto-mechanically generated resonant field enhancement
Source: Sci Rep. 2022 Oct 31;12:18292. doi: 10.1038/s41598-022-22987-3 (PMC9622864; doi:10.1038/s41598-022-22987-3)
Supplement: Supplementary file 1 — Supplementary Information. [file 41598_2022_22987_MOESM1_ESM.pdf]

Opto-mechanically generated resonant field enhancement: supplemental document

In this supplemental information we show explicitly the equations used to obtain the analytic expression for the resonant torque and we review the discrete dipole numerical method used to calculate the torque.

1. ANALYTIC EXPRESSION FOR THE RESONANT TORQUE

The expression for the torque near the resonant condition may be written in a more convenient way by considering the following identities:

$$\Sigma_{j=-N/2}^{j=N/2}\Sigma_{p=1}^{j+N/2}\frac{j}{p}sin(kDp(1+sin(\theta))) = \Sigma_{p=1}^{N}\frac{N-p+1}{2}sin(kDp(1+sin(\theta))) \tag{S1}$$

$$\Sigma_{j=-N/2}^{j=N/2}\Sigma_{p=1}^{j+N/2}\frac{j}{p}cos(kDp(1+sin(\theta))) = \Sigma_{p=1}^{N}\frac{N-p+1}{2}cos(kDp(1+sin(\theta))) \tag{S2}$$

Where summations are given by

$$\Sigma_{p=1}^{N}\frac{N-p+1}{2}sin(kDpf) = \frac{1}{8}csc^2(\frac{kDf}{2})[-sin(kDf(N+1))+(N+1)sin(kDf)] \tag{S3}$$

and

$$\Sigma_{p=1}^{N}\frac{N-p+1}{2}cos(kDpf) = \frac{-1}{8}csc^2(\frac{kDf}{2})[cos(kDf(N+1))+(N+1)cos(kDf)-N-2], \tag{S4}$$

with $f = 1 + sin(\theta)$.
Near the resonant condition we may also approximate

$$(N+1)cos(kDf) - N - 2 \sim -1. \tag{S5}$$

With these approaches we obtain the final expression for the torque.

2. NUMERICAL CALCULATION OF THE TORQUE

We consider each silver nanoparticle as an induced electric dipole. The value of the induced dipole ($\vec{P_n}$) is given by the total field felt by the particle ($\vec{E_n}$) (see eq. (S6)), where ϵ_0 is the permittivity of the vacuum and α the polarizability which, in this study, is a scalar.

$$\vec{P_n} = \epsilon_0 \cdot \alpha \cdot \vec{E_n}\ . \tag{S6}$$

When calculating the total field we must consider the field due to the incident wave plus that emitted by all other particles. The field scattered by a dipole m affecting particle s can be written as in eq. (S7), where $\hat{G}$ is the Green tensor, that depends on the point where the dipole m is placed and on the point of observation ($\vec{r_n}$). $\vec{E_m}$ is the total field over dipole m.

$$\vec{E_{sm}}\,(\vec{r_n}) = \frac{k^2}{\epsilon_0} \cdot \hat{G}(\vec{r_n},\vec{r_m}) \cdot \vec{P_m} = k^2 \cdot \alpha \cdot \hat{G}(\vec{r_n},\vec{r_m}) \cdot \vec{E_m} \tag{S7}$$

In general, the Green tensor is a matrix given by eq. (S8), where $\vec{R} = \vec{r_n} - \vec{r_m}$ and $R = \left\|\vec{R}\right\|$. $\vec{R} \otimes \vec{R}$ is the outer product and $\hat{I}$ is the identity matrix. However, in our case, as the field is polarized in Z and the dipoles are located in the X-Y plane, the Green tensor can be reduced to an scalar (see eq. (S9)).

$$\hat{G}(\vec{r_n},\vec{r_m}) = \frac{e^{ikR}}{4\pi R}\left[\left(1+\frac{ikR-1}{k^2R^2}\right)\cdot\hat{I} + \frac{3-3ikR-k^2R^2}{k^2R^4}\cdot\vec{R}\otimes\vec{R}\right] \tag{S8}$$

$$G_{zz} = \frac{e^{ikR}}{4\pi R}\left(1 + \frac{ikR-1}{k^2R^2}\right) \tag{S9}$$

From this result, eq. (S7) can be reduced to eq. (S10).

$$E_{sm}\left(\vec{r_n}\right) = k^2 \cdot \alpha \cdot G_{zz} \cdot E_m \tag{S10}$$

In the case of multiple particles, the method of coupled dipoles (CDA) can be used. We start by re-writing the scattering problem as a matrix problem (see eq. (S11)). Since $G_{nm} = G_{zz}(r_n, r_m)$, we have:

$$\begin{pmatrix} E_1 \\ E_2 \\ \vdots \\ E_N \end{pmatrix} = \begin{pmatrix} E_{01} \\ E_{02} \\ \vdots \\ E_{0N} \end{pmatrix} + k^2 \cdot \alpha \cdot \begin{pmatrix} G_{11} & G_{12} \cdots & G_{1N} \\ \vdots & \ddots & \vdots \\ G_{N1} & \cdots & G_{NN} \end{pmatrix} \cdot \begin{pmatrix} E_1 \\ E_2 \\ \vdots \\ E_N \end{pmatrix}, \tag{S11}$$

Defining $\tilde{G} = \begin{pmatrix} G_{11} & G_{12} \cdots & G_{1N} \\ \vdots & \ddots & \vdots \\ G_{N1} & \cdots & G_{NN} \end{pmatrix}$ and taking into account that the diagonal elements of the matrix must be zero, we obtain the fields solution as:

$$\begin{pmatrix} E_1 \\ E_2 \\ \vdots \\ E_N \end{pmatrix} = \left[\hat{I} - k^2 \cdot \alpha \cdot \tilde{G}\right]^{-1} \cdot \begin{pmatrix} E_{01} \\ E_{02} \\ \vdots \\ E_{0N} \end{pmatrix} \tag{S12}$$

Once the fields are known, the forces and torques exerted on each particle are calculated. The a component of the electromagnetic force generated on a dipole n is given by

$$F_{an} = \frac{1}{2}\Re\left(P_{xn}\partial_a E^*_{xn} + P_{yn}\partial_a E^*_{yn} + P_{zn}\partial_a E^*_{zn}\right) \tag{S13}$$

Since the field is polarized in z and depends on x and y, the force has only x and y components given by

$$F_{xn} = \frac{1}{2}\Re\left[P_n \partial_x E^*_n\right] \tag{S14}$$

$$F_{yn} = \frac{1}{2}\Re\left[P_n \partial_y E^*_n\right] \tag{S15}$$

Therefore, to calculate the forces, we must first compute the derivatives of the field:

$$\partial_x E^*_{zn} = E_0 \cdot i \cdot k_x \cdot e^{i(k_x \cdot x + k_y \cdot y)} + \sum_{m \neq n} \alpha \cdot k^2 \cdot \left(\partial_x G(\vec{r_n}, \vec{r_m})\right) \cdot E_{zm} \tag{S16}$$

$$\partial_y E^*_{zn} = E_0 \cdot i \cdot k_y \cdot e^{i(k_x \cdot x + k_y \cdot y)} + \sum_{m \neq n} \alpha \cdot k^2 \cdot \left(\partial_y G(\vec{r_n}, \vec{r_m})\right) \cdot E_{zm} \tag{S17}$$

with the derivatives given by

$$\partial_x G(\vec{r_n}, \vec{r_m}) = \left(i - \frac{2}{kR} - \frac{3i}{k^2R^2} + \frac{3}{k^3R^3}\right) \cdot \frac{x_n - x_m}{R} \cdot \frac{ke^{ikR}}{4\pi R} \tag{S18}$$

$$\partial_y G(\vec{r_n}, \vec{r_m}) = \left(i - \frac{2}{kR} - \frac{3i}{k^2R^2} + \frac{3}{k^3R^3}\right) \cdot \frac{y_n - y_m}{R} \cdot \frac{ke^{ikR}}{4\pi R} \tag{S19}$$

Once the forces are known, the total torque on the system with respect to the origin is given by

$$\vec{\tau} = \sum_n \vec{r}_n \times \vec{F}_n = \sum_n \begin{vmatrix} \hat{\imath} & \hat{\jmath} & \hat{k} \\ x_n & y_n & 0 \\ F_{xn} & F_{yn} & 0 \end{vmatrix} = \sum_n \hat{k} \left(x_n F_{yn} - y_n F_{xn} \right), \tag{S20}$$
